# Global Longitudinal Strain in Stress Echocardiography: A Review of Its Diagnostic and Prognostic Role in Noninvasive Cardiac Assessment

**DOI:** 10.3390/diagnostics15162076

**Published:** 2025-08-19

**Authors:** Nikolaos Antoniou, Sotiria Iliopoulou, Dimitrios G. Raptis, Orestis Grammenos, Maria Kalaitzoglou, Marianthi Chrysikou, Christos Mantzios, Panagiotis Theodorou, Ioannis Bostanitis, Dafni Charisopoulou, George Koulaouzidis

**Affiliations:** 1 Cardiology Department, General Hospital George Papanikolaou, 57010 Thessaloniki, Greece; sotiria.ili26@gmail.com (S.I.); raptisdg@gmail.com (D.G.R.); orestes1313@hotmail.com (O.G.); mariakalaitzo25@gmail.com (M.K.); pantheod80@hotmail.com (P.T.); bostangiannis@yahoo.gr (I.B.); 2 Paediatric Department, General Hospital George Gennimatas, 54635 Thessaloniki, Greece; chrysikoumarianthi487@gmail.com; 3 Cardiology Department, AHEPA University Hospital, 54636 Thessaloniki, Greece; chrismantzios1994@gmail.com; 4 Paediatric Cardiology Department, Great Ormond Street Hospital, London WC1N 3JH, UK; dafnithess@yahoo.co.uk; 5 Department of Biochemical Sciences, Pomeranian Medical University, 70-204 Szczecin, Poland

**Keywords:** global longitudinal strain (GLS), stress echocardiography (SE), speckle-tracking echocardiography (STE), coronary artery disease (CAD), myocardial deformation, risk stratification, non-invasive cardiac imaging, longitudinal strain reserve (LSR), myocardial work, subclinical dysfunction

## Abstract

**Background:** The integration of global longitudinal strain (GLS) with stress echocardiography (SE) represents a significant advancement in non-invasive cardiac diagnostics, particularly in the evaluation of coronary artery disease (CAD). GLS, derived from speckle-tracking echocardiography, quantifies myocardial deformation and offers superior sensitivity for detecting subclinical myocardial dysfunction compared to conventional metrics like wall motion and ejection fraction. Recent studies have validated the prognostic and diagnostic efficacy of GLS both at rest and during stress, notably enhancing the detection of obstructive and non-obstructive CAD, microvascular dysfunction, and other cardiac pathologies. **Methods:** This manuscript synthesizes extensive clinical data demonstrating the added value of GLS during stress echocardiography across diverse cardiac conditions—including valvular heart disease, heart failure, cardio-oncology, and pediatric cardiology. Novel metrics like longitudinal strain reserve (LSR), myocardial work indices, and post-systolic strain have further enriched risk stratification strategies. **Results:** The combination of GLS with SE has been shown to approximate the accuracy of invasive coronary angiography in intermediate-risk patients and in cases with equivocal traditional SE findings. Despite its clinical promise, the utility of GLS is challenged by technical limitations, including image quality dependency, inter-vendor variability, and limited applicability during high heart rate states. **Conclusions:** As technological refinement and standardization progress, GLS integrated with SE is poised to become a mainstay in precision cardiology, improving diagnostic yield, guiding therapeutic decisions, and enhancing patient outcomes.

## 1. Introduction

Stress echo (SE) is a tool for the detection and stratification of patients who have history of coronary artery disease (CAD) or those suspected of CAD, with many benefits as it is easily accessible, frequently used, and has low cost [1]. A key component of SE is the visual assessment of wall motion (WM), which is the main characteristic for the detection of ischemia [2]. Despite the fact that expert interpretation of wall motion scoring (WMS) has demonstrated high diagnostic accuracy for CAD, it is inherently subjective and can lead to inter-observer and inter-center variability [2].

The detection of wall motion abnormalities (WMAs) usually occurs later, during the stress echo, as it is a late manifestation in the ischemic cascade. As a result, there are difficulties in the detection of WMA, because of the suboptimal imaging window, the rapid heart rate normalization after stress, and the observer’s skills [3,4]. These challenges may reduce the overall sensitivity and diagnostic accuracy of SE compared to other non-invasive imaging techniques [3,4].

**Figure 1:**
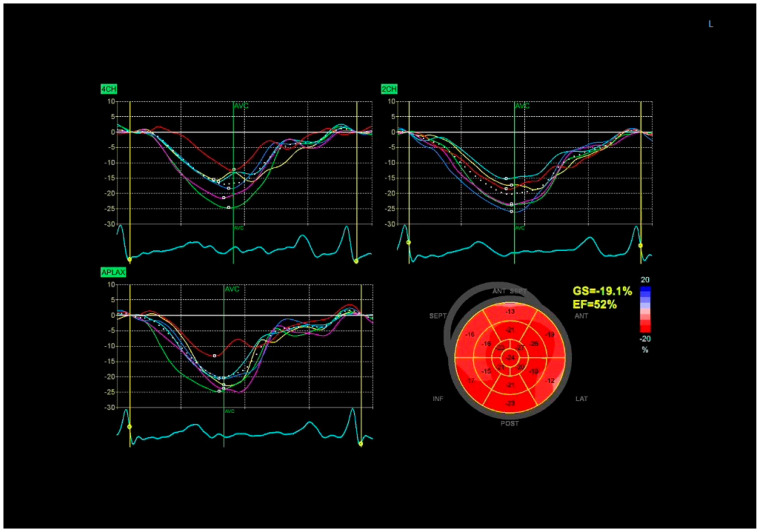
Global longitudinal strain (GLS) in normal left ventricular systolic function. Speckle-tracking echocardiography from apical views shows uniform strain curves with preserved segmental deformation. The bull’s-eye plot demonstrates normal GLS (−19.1%) and ejection fraction (52%).

There are indications that the assessment of left ventricular ejection fraction (LVEF) and global longitudinal strain (GLS) may offer comparable accuracy to WMS in the evaluation of CAD during SE [5] (Figure 1). However, despite their potential, quantitative metrics like GLS have not been widely integrated into routine clinical practice, partly due to their reduced reliability under stress-induced tachycardia and technical demands associated with speckle-tracking analysis [6].

**Figure 2:**
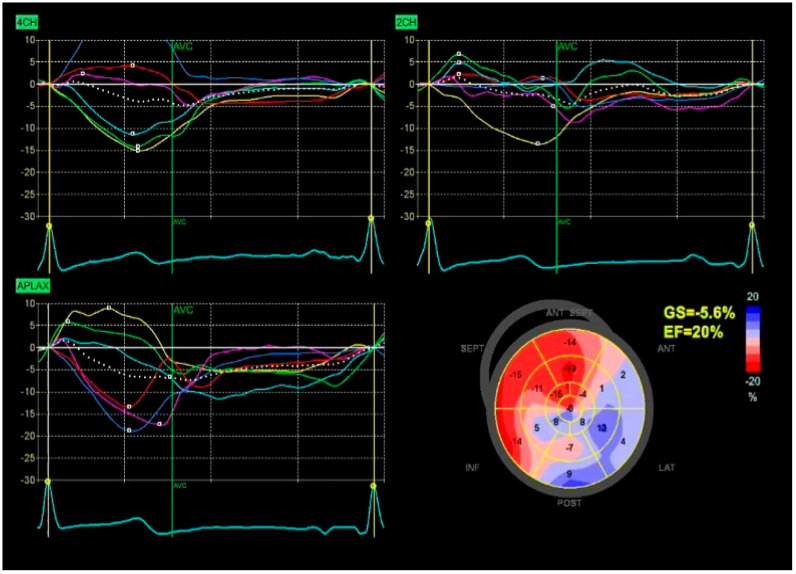
Global longitudinal strain (GLS) analysis in a patient with severe left ventricular dysfunction. Speckle-tracking echocardiography from apical views shows markedly reduced strain in most myocardial segments. The bull’s-eye plot highlights global impairment (GLS –5.6%**,** normal ≤ –18%), with predominant septal and inferior segment involvement. Calculated EF is 20%**,** consistent with advanced systolic failure.

An accurate method which is not affected by the subjectivity of the observer is the longitudinal strain analysis (LS). Some of the benefits of this method are the detection of regional dysfunction earlier in the ischemic cascade, the differentiation of passive motion from true contractile impairment, and the quantification subtle myocardial changes [6,7] (Figure 2). The detection of severe CAD using LS was explored by a few studies.

The clinical utility of GLS during stress echocardiography is partly limited by the lack of standardized reference values, particularly in healthy individuals. While resting GLS in healthy adults typically ranges from –18% to –22%, data on normal GLS values during stress echocardiography are sparse [8]. Donal et al. suggest that GLS in healthy individuals may increase by 2–4% during low-dose dobutamine stress, reflecting enhanced myocardial contractility, but high heart rates and suboptimal imaging windows can reduce measurement reliability [9]. Establishing standardized GLS reference ranges under stress conditions remains an unmet need to enhance the diagnostic accuracy of STE and SE across cardiovascular diseases.

Emerging technologies such as artificial intelligence (AI)-based image processing pipelines have recently been developed to automatically contour the LV and calculate LVEF and GLS from SE images, even in the presence of contrast and across a wide range of heart rates. These AI tools eliminate observer variability and have already shown improved precision in detecting subtle cardiac changes in specific populations, [10].

### 1.1. Coronary Artery Disease

A growing body of evidence supports the integration of speckle-tracking echocardiography (STE) and global longitudinal strain (GLS) into stress echocardiography protocols for the evaluation of coronary artery disease (CAD). These advanced imaging tools enhance diagnostic accuracy, improve risk stratification, and help identify subtle myocardial dysfunction undetected by conventional parameters such as wall motion or ejection fraction.

A study conducted by Hwang et al. showed that GLS during recovery proved to be a reliable marker of significative CAD, Table 1 [11]. A study of 44 patients who had both DSE and coronary angiography demonstrated that patients with CAD had significantly reduced GLS compared to those without, with extents −18.0 ± 3.4% and −21.0 ± 1.9%, respectively. These results suggest that despite apparently normalizing wall motion after stress, left ventricular function may remain depressed, arguing for the presence of post-ischemic stunning in CAD patients. In support of this point of view, the work of Cognet et al. introduces the concept of longitudinal strain reserve (LSR) that demonstrates the discrepancy between GLS at rest and at peak vasodilation achieved with a pharmacological agent, dipyridamole [12]. In their study of 63 patients with no prior history of CAD, significant differences in LSR measurements were observed, particularly among diabetic patients who displayed a greater strain reserve compared to non-diabetics. These implications are important, as LSR may represent a key parameter for the identification of subclinical contractile dysfunction, with prognostic value in older persons and in persons with diabetes in particular.

Also, Montgomery et al. demonstrated the value of resting GLS for the detection of non-occlusive CAD, Table 1 [13]. In their analysis of 123 patients, patients with clinically significant stenoses (≥50%) were found to have significantly lower baseline GLS. The above is in agreement with those of previous studies in which the diagnostic accuracy of GLS at rest was equal to the wall motion score index from stress testing; that is, resting GLS might identify subclinical myocardial dysfunction not visible in patients with silent CAD. These findings reveal the value of GLS as an adjunctive measure in the diagnosis of CAD, especially with negative findings in conventional stress echo.

Gaibazzi et al. have established the predictive capabilities of GLS [14]. On their study, a cutoff of GLS > −20.7% was significant to predict CAD, with an area under the curve (AUC) of 0.86 in patients undergoing SE. This adds further supporting information on the ability of GLS to improve the detection of clinically relevant CAD during stress testing.

Rumbinaitė et al. demonstrated the superiority of GLS over diastolic strain rate in their study that included 127 patients with moderate to high pretest probability of CAD, Table 1 [15]. They concluded with the results that the peak values of GLS were lower in CAD-positive subjects and reassured the diagnostic accuracy of WAL with AUC values achieved of more than 0.95. These details highlight the importance of GLS for the discrimination of CAD and non-CAD etiologies and, as such, in clinical decision-making.

Complementing studies including that by Cusmà-Piccione et al. demonstrate the strong value of GLS over wall motion analysis, Table 1 [16]. Their results demonstrated that the sensitivity of wall motion analysis for detecting single-vessel CAD was relatively low (44–55%), whereas GLS variables (GLS during dipyridamole stress) increased the sensitivity to 84% and specificity to 92%. This indicates that the inclusion of advanced strain imaging techniques can substantially improve diagnostic performance in echo labs with poor contrast resolution using traditional methods.

In CAD patients with low LVEF, GLS combined with SE is particularly valuable for assessing myocardial viability and residual ischemia, guiding revascularization decisions. In this high-risk group, reduced LVEF often reflects ischemic or hibernating myocardium, and GLS can detect subtle improvements in myocardial deformation during low-dose dobutamine stress, indicating viable tissue amenable to revascularization. For instance, studies have shown that an increase in GLS (ΔGLS) during DSE predicts functional recovery post-revascularization, with higher sensitivity than wall motion score index (WMSI) for identifying viable myocardium [17,18]. Additionally, GLS can quantify contractile reserve, helping differentiate ischemic from non-ischemic causes of LV dysfunction [17]. This approach enhances risk stratification and supports clinical decisions regarding coronary artery bypass grafting (CABG) or percutaneous coronary intervention (PCI) in patients with low LVEF, where conventional SE may be less reliable due to baseline wall motion abnormalities [18].

The application of STE combined with physical exercise stress echocardiography in CHD offers diagnostic potential but faces unique challenges. Exercise stress, such as treadmill or bicycle testing, is widely used to provoke ischemia in CHD patients with preserved functional capacity. Karlsen et al. demonstrated that post-exercise GLS increases in patients without CAD (AUC 0.97, sensitivity 93.9%, specificity 93.2%), while remaining blunted in those with CHD, outperforming LVEF and WMSI in suspected unstable angina, Table 1 [19]. Similarly, Mansour et al. found that GLS at peak exercise enhanced chest pain evaluation, with GLS ≥ –20% ruling out obstructive CAD, Table 1 [20]. However, high heart rates (>120–130 bpm) and poor acoustic windows due to patient positioning or respiratory motion limit GLS feasibility during peak exercise, reducing its reliability compared to pharmacological stress [21]. Despite these challenges, STE with exercise stress remains a valuable tool for detecting inducible ischemia in CHD, particularly when image quality is optimized with contrast enhancement, Table 1 [22].

Ejlersen et al. have shown the importance of this utility of GLS in more nuanced contexts, focusing on layer-specific GLS during adenosine stress echocardiography, Table 1 [23]. The authors of this study found even lower stress-induced changes in GLS in CAD patients, further suggesting that enhanced strain analysis could provide incremental diagnostic value in a key population in which traditional diagnostic methods can underperform.

Likewise, Dattilo et al. investigated whether 2D strain offers superior predictive power in the context of non-diagnostic stress echocardiography, Table 1 [24]. Their findings support the use of GLS as a diagnostic parameter, with a ΔGLS ≤0% being predictive of CAD. These results support incorporating GLS into routine clinical practice and stress testing.

The diagnostic utility of combining DSE with STE was further validated in the work of Smiianov et al. who showed that while individual parameters like GLS or wall motion score index (WMSI) have moderate diagnostic value, their combined interpretation closely mirrors the accuracy of coronary angiography, Table 1 [25]. This integrative approach improved clinical decision-making, particularly in borderline or inconclusive cases.

Strain imaging has also shown superior performance in lesion localization. Roushdy et al. and Ilardi et al. both found that global and regional longitudinal strain significantly improved detection of left anterior descending (LAD) artery disease, offering better concordance with angiography than wall motion analysis alone, Table 1 [26,27]. Notably, strain parameters reduced false positives in non-LAD territories, enhancing both sensitivity and specificity.

Elamragy et al. further confirmed that peak GLS during DSE improved diagnostic performance in patients with intermediate CAD risk, Table 1 [28]. When combined with conventional stress markers, strain imaging yielded better sensitivity and negative predictive value without compromising specificity.

In dipyridamole stress echocardiography, Licordari et al. showed that GLS effectively detected mild CAD, even in cases with inconclusive traditional test results [29]. Reduced GLS at baseline identified subclinical myocardial dysfunction, and despite stable values over five years, the initial reduction carried prognostic relevance.

Ragab et al. introduced the post-systolic strain index (PSI) as an additional diagnostic metric during the recovery phase of DSE, which proved particularly sensitive in revealing subtle ischemia often missed at peak stress, advocating for its incorporation into standard practice, Table 1 [30].

**Table 1:** Clinical characteristics, study protocol, and results of the included studies. GLS: global longitudinal strain, ICA: invasive coronary angiography, SE: stress echocardiogram, ESE: exercise stress echocardiogram, DSE: dobutamine stress echocardiogram, WMSi: wall motion score index, WMA: wall motion abnormalities, CTCA: coronary CT angiography, ΔGLS: percentage of variation in GLS, CAD: coronary artery disease, AFI: Automated Functional Imaging, SR: strain rate, CMR: Cardiac MRI, RLS: regional longitudinal strain, GCS: global circumferential strain.

Author	Year/Country	Type of the Study	Number of Patients	Type of Stress	Echocardiographic Parameters	Anatomical Test	Results
Hwang [11]	2014/Korea	Prospective	44	DSE	GLS (rest and recovery); WMA (peak stress)	ICA	GLS at recovery: Sens. 71%, Spec. 83%; WMA at peak stress: Sens. 72%, Spec. 85%
Montgomery [13]	2011/USA	Retrospective	123	DSE	GLS (rest); WMSi (rest and peak stress)	ICA	GLS predicted coronary stenosis ≥50%; AUC: ~0.72; comparable to WMSI. Global strain cut point value of ~17.77% sensitivity/specificity (66/76%)
Rumbinaitė [15]	2016/Lithuania	Prospective	127	DSE	GLS (rest); GLS (low to high dose); WMA (rest); WMA (low to high dose); Diastolic SR	ICA + stress CMR	Stress GLS best CAD predictor (AUC: 0.955; Sens. 94%, Spec. 92%) Combination of WMA and GLS at rest AUC: 0.951; Sens. 93%, Spec. 87% (*p* < 0.001); combination of WMA and GLS at high dobutamine dose AUC: 0.977; Sens. 96%, Spec. 93% (*p* < 0.001)
Cusmà-Piccione [16]	2015/Italy	Prospective	52	Dipyridamole SE	GLS (rest and peak stress) WMSi (rest & peak stress)	ICA	GLS more accurate than wall motion for single-vessel CAD (Sens. 84%, Spec. 92% vs. Sens. 44%, Spec. 51%)
Ejlersen [23]	2016/Denmark	Prospective	132	Adenosine SE	Layer-specific GLS AFI	ICA	ΔGLS (endo/mid/epi) predicted CAD with AUC: ~0.8. From the cut of values: ΔendoGLS: Sens. 65%, Spec. 85%; ΔmvGLS: Sens. 59%, Spec. 91%; ΔepiGLS: Sens. 59%, Spec. 84%; ΔAFI: Sens. 54%, Spec. 80%
Dattilo [24]	2016/Italy	Prospective	90	Dipyridamole SE	ΔGLS (rest and peak stress)	CTCA	ΔGLS ≤ 0%: Sens. 95%, Spec. 93%; AUC: 0.91 for coronary stenosis 15–50%)
Mansour [20]	2018/Lebanon	Prospective	103	ESE	GLS (rest and peak stress)	CTCA	pGLS ≥ 20% ruled out obstructive CAD
Smiianov [25]	2020/Ukraine	Prospective	140	DSE	GLS; ΔGLS; ΔWMSI	ICA	DSE with GLS had Sens. 98.3%, Spec. 91.7%, AUC = 0.98. Combined ΔGLS + WMSI was less accurate (Sens. 86.2%, Spec. 80.4%, AUC 0.83)
Roushdy [26]	2017/Egypt	Prospective	80	DSE	2D GLS, GCS, territorial strain	ICA	Peak GLS (cutoff –16.75) showed Sens. 77.4%, Spec. 83.3%; better agreement than WMSI for lesion detection, vessel number, and CAD localization
Ilardi [27]	2021/Italy	Prospective	50	DSE	GLS, RLS (peak stress)		GLS and RLS more accurate than WMSI in detecting LAD stenosis (94.3% accuracy); less effective for LCX/RCA; combination RLS + WMSI improved LAD detection
Elamragy [28]	2020/Egypt	Prospective	101	DSE	GLS (peak stress)	ICA	GLS increased diagnostic accuracy in intermediate-risk CAD patients; combining GLS cutoff with DSE had higher AUC (0.9, *p* < 0.001): Sens. 95.9% and Spec. 84.6%
Licordari [29]	2022/Italy	Longitudinal	65	Dipyridamole SE	GLS (rest and peak stress) 2D strain	CTCA	GLS predicted outcome in early CAD (5-year follow-up). Left ventricular GLS improves the accuracy of SE in the detection of mild CAD
Ragab et al. [30]	2025/Egypt	Prospective	125	DSE	GLS (rest, peak stress and recovery)	ICA	Adding GLS to DSE improved CAD detection. GLS at recovery Sens. 95% and Spec. 98%
Abazid [22]	2024/Canada	Prospective	33	ESE	GLS (rest and peak stress)	ICA	GLS improved diagnostic performance for ischemia, AUC = 0.72; a cutoff value of -20% of stress LS Sens. 71% and Spec. 60% for ruling out inducible myocardial ischemia (*p* < 0.0001)
Karlsen [19]	2022/Norway	Prospective	78	ESE	GLS (rest and recovery) WMSi (rest & recovery)	ICA + FFR	Post-exercise GLS increase ruled out CAD (AUC = 0.97) with Sens. 93.9% and Spec. 93.2%; superior to LVEF and WMSi
Davis [31]	2024/USA	Prospective	120	ESE	GLS	CTCA	GLS predicted inducible ischemia in patients with no obstructive CAD

However, the utility of GLS is more limited in patients with ischemia and no obstructive coronary artery disease (INOCA). Davis et al. reported no meaningful correlation between resting or dynamic GLS and ischemic burden in these patients, suggesting strain imaging may not adequately capture subclinical dysfunction in this population, Table 1 [31].

In patients recovering from acute coronary syndrome (ACS), GLS combined with SE provides valuable prognostic insights for predicting adverse cardiovascular events. Post-ACS, residual myocardial dysfunction and ischemia increase the risk of reinfarction, heart failure, or mortality. Eitel et al. have shown that resting GLS measured shortly after ACS is a strong predictor of major adverse cardiac events (MACEs), with GLS < −15% associated with a hazard ratio of 2.5 for MACE over 2 years [32]. Furthermore, SE with GLS assessment can identify residual ischemia or reduced contractile reserve, guiding secondary prevention strategies. For instance, a blunted ΔGLS during low-dose dobutamine stress post-ACS indicates impaired myocardial recovery and higher risk of adverse outcomes [33]. Integrating GLS with SE in post-ACS patients enhances risk stratification, supporting tailored management to prevent recurrent events.

Lech et al. studied the exercise-induced differences in GLS in 50 patients with asymptomatic severe aortic stenosis (ASAS), and LVEF ≥ 55%, Table 2 [34]. Both groups—ASAS patients and healthy subjects—showed an increase in GLS during exercise in the present study. However, the ΔGLS was less in the ASAS group, compared to the healthy controls (–0.8 ± 3.0% vs. –2.2 ± 3.1%, *p* = 0.086). This discrepancy may imply abnormality of contractile reserve in the ASAS group supporting the potential value of GLS as an early parameter of myocardial dysfunction before a significant reduction in LVEF occurs. The results suggest that morphological changes caused by hypertrophy can have an impact on myocardial fiber function despite the lack of overt signs, and notable reduction in EF, which GLS can detect.

Dahou et al. conducted a total analysis of 116 low-flow, low-gradient AS patients with low LVEF. Their objective was to assess the prognostic value of resting and DSE GLS, Table 2 [35]. The study showed that resting and stress GLS values were both significant independent predictors of all-cause death, indicative of the utility of these measures as important markers for risk stratification. Patients with stress GLS < 10% had poor survival compared with the other groups over three years (30% vs. 74%, *p* = 0.001). Most importantly, these results suggest that detection of GLS, especially under stress conditions, is superior to identification of conventional echocardiographic indices, such as LVEF or stroke volume, in the prognostically guided treatment of high-risk patients in this population.

The study by Arbucci et al. builds upon the theory of contractile reserve estimated by GLS (CR-GLS) in asymptomatic severe aortic stenosis, Table 2 [36]. Their data showed that those without CR-GLS had worse clinical profiles and higher prevalence of major cardiac events compared with those with preserved CR-GLS. This highlights the clinical importance of CR-GLS as a superior and sensitive parameter for early identification of myocardial dysfunction compared to CR obtained by conventional measures of ejection fraction.

In the context of aortic regurgitation, D’Andrea et al. focused on identifying subclinical functional impairments in asymptomatic patients with severe AR, Table 2 [37]. Their study illustrated that, despite having preserved resting LVEF, patients exhibited significant reductions in myocardial deformation and efficiency when compared to healthy controls. The researchers found that impairments in GLS were associated with diminished contractile reserve and increased pulmonary congestion signs, further cementing the role of GLS in assessing myocardial work efficiency and subclinical dysfunction.

Li Q et al. reviewed the prognostic role of combining STE with DSE in patients with chronic severe aortic regurgitation and markedly reduced LVEF undergoing surgical aortic valve replacement, Table 2 [38]. Among echocardiographic parameters, GLS, at rest and during stress, emerged as strong independent predictors of postoperative improvement. This combined imaging approach was shown to enhance risk stratification and guide surgical decision-making in high-risk patients with impaired LV function.

For mitral valve disease, particularly mitral regurgitation (MR), GLS combined with SE is emerging as a valuable tool for detecting subclinical LV dysfunction and guiding early intervention, such as mitral valve repair. In asymptomatic primary MR with preserved LVEF, GLS at rest can identify early myocardial impairment, while SE unravels reduced contractile reserve, which is critical for timing valve repair before irreversible LV dysfunction develops. Lancellotti et al. have shown that a reduced GLS (< −18%) in MR patients is associated with worse outcomes, while exercise induced changes in GLS during SE can predict postoperative LV dysfunction, aiding in early qualification for repair [39]. This approach enhances risk stratification by identifying patients at risk of progression despite normal LVEF, supporting guideline recommendations for earlier intervention in severe MR. Thus, GLS and SE offer complementary diagnostic and prognostic insights across both aortic and mitral valve diseases, improving the precision of clinical decision-making.

### 1.2. Chronic Kidney Disease

In their 2024 study, Tsartsalis et al. investigated the prognostic value of resting myocardial deformation parameters in predicting ischemic responses during DSE in patients with end-stage chronic kidney disease (CKD), Table 2 [40]. The study revealed that, while most patients exhibited preserved or mildly reduced LV systolic function, a significant subset displayed impaired left atrial (LA) strain patterns. Reduced LA strain reflects compromised LA function, often resulting from increased LV filling pressures due to ischemic ventricular myocardium, which impairs LV diastolic function. In CKD, where fluid overload and microvascular dysfunction are prevalent, these changes can manifest as subclinical ischemia. The study found that both reduced GLS of the LV and impaired LA strain at rest were independent predictors of inducible myocardial ischemia during DSE. These findings highlight the complementary role of LA strain as an early, indirect marker of ventricular ischemia, enhancing the detection of subclinical cardiac dysfunction in CKD patients when used alongside LV GLS.

### 1.3. Liver Failure

Cardiovascular assessment in patients with end-stage liver disease (ESLD) presents unique challenges, as traditional methods like wall motion analysis during stress echocardiography often lack sensitivity in this population. In a pivotal study by Anderson et al. the incorporation of STE into DSE significantly improved the diagnostic performance for detecting CAD in ESLD patients, Table 2 [41]. While conventional wall motion analysis demonstrated limited sensitivity, strain-derived parameters—particularly GLS and the post-systolic shortening index (PSSi)—offered substantial diagnostic enhancement. Among these, PSSi emerged as the most balanced and reliable metric, offering superior sensitivity and specificity for CAD detection. These findings underscore the clinical value of integrating strain analysis, and especially PSSi, into standard DSE protocols for improved cardiovascular risk stratification in ESLD.

Building on this, Zamirian et al. investigated myocardial reserve in cirrhotic patients using dobutamine stress echocardiography with both speckle-tracking and tissue Doppler imaging, Table 2 [42]. Despite normal systolic function at rest, cirrhotic patients demonstrated a blunted contractile response to stress, with inadequate increases in GLS and strain rate compared to healthy controls. This diminished myocardial reserve, often invisible during resting evaluation, reflects subclinical cardiac dysfunction characteristic of cirrhotic cardiomyopathy. The inability of the heart to augment its function under physiological stress contributes to the heightened vulnerability of these patients to heart failure and hemodynamic collapse during surgery, infection, or fluid shifts.

### 1.4. Cardio-Oncology

Khouri et al. conducted a study involving 57 patients with breast adenocarcinoma and preserved LVEF, all of whom had previously received standard-dose doxorubicin-containing chemotherapy, Table 2 [43]. The primary aim was to identify subclinical myocardial dysfunction using a combination of resting three-dimensional echocardiography (3DE), GLS, and exercise stress echocardiography. The 3DE assessment revealed that patients had significantly lower LVEF compared to controls (55 ± 4% vs. 59 ± 5%; *p* = 0.005), a difference not detected by conventional 2D echocardiography. Despite preserved LVEF, resting GLS identified reduced myocardial systolic performance in 20% of patients. Furthermore, during exercise stress testing, patients showed a 12% reduction in left ventricular stroke volume and a 24% decrease in cardiac index compared to controls. These findings highlight that GLS, particularly when used alongside stress echocardiography protocols, serves as a sensitive tool for detecting early cardiac dysfunction in oncologic patients—even when resting echocardiographic parameters appear normal.

A prospective study by Nabiałek-Trojanowska et al. investigated early, asymptomatic cardiac dysfunction in survivors of mediastinal lymphoma treated with chemotherapy, with or without radiotherapy, Table 2 [44]. While resting echocardiography revealed generally preserved LVEF, borderline GLS, and reduced stroke volume—with minimal differences between treatment groups—these findings did not fully capture subclinical myocardial impairment. DSE, however, revealed significant reductions in left ventricular contractile reserve (LVCR), detectable through changes in LVEF and GLS, and universally impaired when assessed using the Force parameter. This highlights the diagnostic value of combining GLS with DSE to identify subclinical cardiotoxicity not evident at rest.

### 1.5. Hypertrophic Cardiomyopathy

In a prospective trial, Reant et al. followed 115 patients with hypertrophic cardiomyopathy (HCM) for 19 months to determine predictors for cardiac events, Table 2 [45]. The GLS was significantly lower in patients with adverse events, as the study revealed. A resting GLS ≤15% was an independent predictor of outcome (hazard ratio [HR] 3.84, *p* = 0.017), as was an exercise-induced peak left ventricular outflow tract (LVOT) gradient ≥50 mmHg (HR 3.29, *p* = 0.028). These results demonstrate the incremental value of resting GLS associated with exercise-induced hemodynamics in risk stratification of HCM patients.

### 1.6. Heart Transplant

A pilot study by Cameli et al. explored the diagnostic utility of GLS in evaluating marginal heart donors, Table 2 [46]. Dipyridamole stress echocardiography was performed on 13 potential donors, and the change in GLS (ΔGLS) was used to differentiate transplant eligibility. Hearts deemed suitable for transplantation showed a significant increase in GLS (+13.2%), while those deemed ineligible demonstrated a decrease (−6.1%; *p* = 0.0001). These findings suggest that strain imaging, particularly ΔGLS during pharmacologic stress, may serve as a reliable and objective tool to reduce operator dependency in the assessment of donor heart viability using STE.

### 1.7. Heart Failure with Reduced Ejection Fraction

A prospective study by Paraskevaidis et al. evaluated the prognostic significance of multidimensional myocardial deformation, including longitudinal, radial, and circumferential strain, during DSE in 100 patients with heart failure with reduced ejection fraction (HFrEF), followed for a median of 4 years to assess cardiac mortality, Table 2 [47]. During follow-up, 26% of patients experienced cardiac death. The analysis identified changes in global longitudinal strain (ΔGLS) and radial strain at rest as the strongest independent predictors of cardiac mortality (*p* = 0.022). Patients with impairments in both parameters exhibited the poorest prognosis. The addition of regional strain imaging, particularly radial strain, to GLS enhances risk stratification by capturing localized myocardial dysfunction, which is often heterogeneous in HFrEF due to ischemic or fibrotic changes. We recommend incorporating regional strain, especially radial strain, alongside GLS in DSE protocols for HFrEF patients to improve the detection of subclinical dysfunction and guide prognostic assessment.

### 1.8. Childhood and Congenital Heart Disease

Broberg et al. demonstrated that combining GLS with DSE is an effective strategy for detecting early cardiac dysfunction in childhood cancer survivors treated with anthracyclines, Table 2 [48]. While LVEF often appears normal at rest, impaired myocardial contractile reserve can be unmasked through strain analysis during stress testing. This strategy enhances early identification of at-risk pediatric patients and supports more personalized, long-term cardiac surveillance.

Taha et al. assessed right ventricular (RV) function in children following the Senning atrial switch procedure, a surgery used to treat transposition of the great arteries, Table 2 [49]. Traditional measures revealed limited contractile reserve both at rest and during stress. However, GLS assessed during DSE uncovered hidden RV contractile reserve in a significant number of patients. This strain-based assessment helped guide the continuation of anti-fibrotic treatment, highlighting its value in monitoring right ventricular performance in this population.

**Table 2:** Clinical characteristics, study protocol and results of the included studies. Dobutamine stress echocardiogram, ICA: invasive coronary angiography, LVEF: left ventricular ejection fraction, HCM: hypertrophic cardiomyopathy, AUC: area under the curve, WMSI: wall motion score index, WM: wall motion, LVOT: left ventricular outflow tract, AS: aortic stenosis, ASAS: asymptomatic severe aortic stenosis, ΔGLS: percentage of variation in GLS, HFrEF: heart failure with reduced ejection fraction, CAD: coronary artery disease, RS: radial strain, CS: circumferential strain, TFO: Tetralogy of Fallot, LSR: longitudinal strain reserve.

Author	Year/Country	Type of the Study	Number of Patients	Type of Stress	Echocardiographic Parameters	Results
Cognet [12]	2013/France	Prospective	63 non-ischemic patients	Dipyridamole SE	GLS; LSR	LSR > 0% predicted mortality; LSR higher in diabetics, lower with age
Gaibazzi [14]	2014/Italy	Retrospective	100 patients referred for CA	Dipyridamole SE	GLS (rest/peak stress); WM (rest/peak stress)	GLS > –20.7% predicted CAD, AUC 0.86; stress GLS superior for demonstration of reversible ischemia to rest GLS
Licordari [29]	2022/Italy	Longitudinal	n/a	Not specified	2D strain	2D strain predicted outcome in early ischemic heart disease (5-year follow-up)
Lech [34]	2017/Poland	Observational	50 ASAS and 21 control patients	ESE	GLS (rest/peak stress); ∆GLS	ASAS patients had lower ∆GLS vs. controls (–0.8% vs. –2.2%); stress GLS indicates preserved functional reserve in ASAS
Dahou [35]	2015/Canada	Prospective	75 with low-flow, low-gradient AS	DSE	GLS (rest/peak stress)	Stress GLS <10% predicted poor survival; stress GLS is superior to rest GLS
Arbucci [36]	2022/Argentina	Prospective	101	Not specified	GLS	Contractile reserve via GLS predicted long-term outcome in asymptomatic AS
D’Andrea [37]	2020/Italy	Prospective	170	ESE	GLS; Myocardial Work	SESAR protocol revealed LV contractile reserve in asymptomatic severe AR
Li [38]	2023/China	Prospective	50	DSE	GLS	Combined GLS and DSE predicted surgical outcome in severe AR
Tsartsalis [40]	2024/Greece	Prospective	61	Resting	GLS (rest)	Resting GLS identified ischemia in CKD patients
Anderson [41]	2023/USA	Prospective	36	DSE	GLS; Post-systolic Shortening (PSS)	GLS + PSS improved sensitivity of DSE in end-stage liver disease
Zamirian [42]	2019/Iran	Prospective	30	DSE	GLS; TDI	Reduced myocardial reserve in cirrhotic patients
Khouri [43]	2014/USA	Prospective	53 breast cancer survivors	ESE	GLS; 2D LVEF; 3D LVEF	GLS identified dysfunction in 20% with preserved EF; correlated with VO_2_peak
Nabiałek-Trojanowska [44]	2023/Poland	Observational	60	None (rest echo)	GLS	Asymptomatic lymphoma survivors showed subtle GLS abnormalities
Reant [45]	2015/France	Prospective	115 with HCM	ESE	GLS (rest/peak stress); LVOT gradient (rest/peak)	GLS ≤15% and LVOT ≥50 mmHg predicted adverse events
Cameli [46]	2016/Italy	Observational	13 marginal donors	Dipyridamole SE	GLS (rest/peak stress); ΔGLS (rest/peak streess)	ΔGLS identified transplantable hearts; ΔGLS normal and pathological stress echo (+ 13.2 ± 5.2 vs. −6.1% ± 3.1%, *p* = 0.0001)
Paraskevaidis [47]	2017/Greece	Prospective	100 HFrEF patients	DSE	GLS; RS; CS	Stress ΔGLS and rest RS independent predictors of long-term cardiac mortality
Broberg [48]	2023/Sweden	Prospective	152	DSE	GLS	Childhood cancer survivors had reduced myocardial contractile reserve
Taha [49]	2020/Egypt	Prospective	61	DSE	GLS (systemic RV)	Quantified contractile reserve in post-Senning children
Mese [50]	2016/Turkey	Prospective	20 adolescents with repaired TOF & 20 controls	DSE	GLS LV and RV (rest/stress); GCS LV and RV (rest/stress)	Stress GLS superior to rest GLS: TOF patients had blunted GLS response; revealed early LV dysfunction

## 2. Discussion

This study adds to the increasing evidence for incorporating strain imaging, in particular, GLS, into standard SE protocols in order to enhance diagnostic accuracy and risk stratification in patients with CAD. Conventional SE, based on the recognition of RWMA, has been the established reference standard for detecting induced ischemia. But its sensitivity is limited in subclinical dysfunction, non-obstructive atherosclerosis, or microvascular disease. Our results are consistent with the recent literature indicating that the incorporation of GLS has the ability to offer a more complete and earlier identification of myocardial dysfunction.

Multiple studies have emphasized the diagnostic potential of GLS at both rest and during pharmacologic stress. Hwang et al. showed that abnormal GLS in the post-dobutamine recovery phase was a powerful indicator of significant CAD, despite the presence of apparently normalized wall motion [11]. Montgomery et al. also showed that GLS at rest could discriminate between patients with and without angiographically significant stenoses, supporting the utility of strain imaging in cases of silent or non-occlusive CAD [13]. In line with these findings, Rumbinaitė et al. and Gaibazzi et al. found that GLS exhibited comparable, and in some cases superior, diagnostic accuracy to wall motion score index, even yielding area-under-the-curve (AUC) values greater than 0.95 [14,15].

The use of SE and GLS in combination seem to have a synergistic diagnostic value, especially in intermediate-risk subjects and whenever the findings of conventional stress echo are equivocal or uncertain. Smiianov et al. and Elamragy et al. showed that the addition of GLS to SE protocols significantly increased the sensitivity and specificity of the tests, reaching close to angiographic diagnostic performance [25,28]. This integrated approach further allows better localization of the lesion. Regional and global strain results have shown better correlation with angiographic findings (in particular, those of the left anterior descending (LAD) artery) and reducing false positives in non-LAD territories.

Another advantage of GLS is its ability to detect subtle myocardial dysfunction in several clinical scenarios other than CAD. For example, patients with chronic kidney disease, liver disease, and cardio-oncology cohorts may demonstrate preserved ejection fraction but abnormal GLS at rest or with stress, suggestive of subtle dysfunction. Collectively, myocardial work indices derived from strain imaging (e.g., GWI and GCW) demonstrated an added value for detecting coronary microvascular dysfunction in the presence of otherwise normal findings. These indices improve the diagnostic capability of echocardiography, which is not limited to the classical visual RWMA search.

Despite these strengths, there are critical limitations to strain imaging that need to be recognized. GLS analysis is (very) dependent of optimal image quality and accuracy may be impaired in poor acoustic window patients and when HR is >120–130 beats per minute, such as maximum treadmill or high-dose dobutamine stress [51]. In addition, vendor-specific differences in the strain measurement algorithms also presents a challenge for inter-institutional reproducibility, but ongoing standardization task forces are addressing this issue. Picano et al. emphasizes that both inter-vendor and intra-vendor variability become more pronounced during stress testing, as changes in image quality, heart rate, loading conditions, and left ventricular (LV) volume can independently affect regional and global strain values [51]. In clinical practice, GLS is estimated to be feasible in approximately 80–90% of cases under optimal conditions, but this rate may decrease in challenging scenarios such as obesity, lung disease, or peak stress with tachycardia [52,53,54]. These factors complicate the accurate assessment of myocardial deformation. To enhance the acquisition of GLS measurements, several strategies could be employed. For instance, employing simplified scan techniques that focus on specific cardiac regions might reduce variability. Furthermore, optimizing the imaging protocol, as proposed in various studies, can facilitate better data acquisition and reliability. Enhancing training for echocardiographers to better navigate the nuances of GLS measurement in real-time clinical settings may help mitigate the limitations encountered during stress testing. Emerging AI-based image processing tools [10] and contrast-enhanced echocardiography [19] are promising solutions to improve GLS feasibility by enhancing endocardial tracking and reducing observer variability, even in suboptimal conditions. These advancements support the integration of GLS into standard SE protocols in clinical laboratories, with ongoing standardization efforts expected to further enhance its applicability. Furthermore, in patients with ischemia and non-obstructive coronary artery disease (INOCA), studies like those by Davis et al. [31] have demonstrated that impaired GLS may not correlate with ischemic burden, highlighting the limitations of strain imaging in diffuse or microvascular diseases.

An important consideration when interpreting GLS values is their dependency on loading conditions. In AS, increased afterload due to outflow obstruction can reduce GLS values, even in the presence of preserved LVEF, as demonstrated by Lech et al., who reported a blunted ΔGLS in asymptomatic severe AS patients compared to controls [34]. This reduction reflects the impact of elevated afterload on myocardial fiber function, which GLS detects earlier than conventional LVEF measurements. Similarly, in HCM, increased afterload from LVOT obstruction, as noted by Reant et al., contributes to reduced GLS (≤15%), which was an independent predictor of adverse cardiac events [45]. These conditions highlight the need to consider loading conditions when interpreting GLS, as changes in preload and afterload can mimic or mask subclinical dysfunction.

Also, although GLS is able to detect early subendocardial ischemia, it should not supplant assessment of RWMA in selective scenarios, and especially not in cases of high intensity physical exertion where strain imaging may not be technically feasible. In such circumstances, SE maintains its diagnostic potential when contrast media are utilized for enhancing endocardial border. In addition, stress echocardiography continues to be in demand on account of its widespread use, low cost, absence of ionizing radiation, and ability to evaluate multiple functional domains including coronary flow reserve and pulmonary congestion through the ABCDE protocol.

## 3. Conclusions

In conclusion, as the methodology of stress echocardiography matures, it is poised to play a pivotal role in the future of cardiology, especially with the integration of advanced imaging techniques and machine learning algorithms to improve diagnostic accuracy and patient outcomes. These advances promise not only to refine existing protocols but also to ensure that clinicians can make more informed decisions regarding patient care in the face of evolving cardiovascular diseases.
